# Glymphatic transport is reduced in rats with spontaneous pituitary tumor

**DOI:** 10.3389/fmed.2023.1189614

**Published:** 2023-08-04

**Authors:** Lian Li, Guangliang Ding, Li Zhang, Hao Luo, Esmaeil Davoodi-Bojd, Qingjiang Li, Michael Chopp, Zheng Gang Zhang, Quan Jiang

**Affiliations:** ^1^Department of Neurology, Henry Ford Health, Detroit, MI, United States; ^2^Department of Physics, Oakland University, Rochester, MI, United States

**Keywords:** glymphatic transport, spontaneous pituitary tumor, rat, MRI, kinetic modeling

## Abstract

**Background and objective:**

Pituitary tumor in patients induces adverse alterations in the brain, accompanied by cognitive deficits. Dysfunction of glymphatic waste clearance results in accumulation of neurotoxic products within the brain, leading to cognitive impairment. However, the status of glymphatic function in the brain with pituitary tumor is unknown. Using magnetic resonance imaging (MRI) and an advanced mathematical modeling, we investigated the changes of glymphatic transport in the rats carrying spontaneous pituitary tumor.

**Methods:**

Rats (22–24 months, female, Wistar) with and without pituitary tumor (*n* = 7/per group) underwent the identical experimental protocol. MRI measurements, including T2-weighted imaging and dynamic 3D T1-weighted imaging with intracisternal administration of contrast agent, were performed on each animal. The contrast-induced enhancement in the circle of Willis and in the glymphatic influx nodes were observed on the dynamic images and verified with time-signal-curves (TSCs). Model-derived parameters of infusion rate and clearance rate that characterize the kinetics of glymphatic tracer transport were evaluated in multiple representative brain regions.

**Results:**

Our imaging data demonstrated a higher incidence of partially enhanced circle of Willis (86 vs. 14%; *p* < 0.033) and a lower incidence of enhancement in glymphatic influx nodes of pituitary (71 vs. 100%) and pineal (57 vs. 86%) recesses in the rats with pituitary tumor than in the rats with normal appearance of pituitary gland, indicating an intensification of impaired peri-vascular pathway and impeded glymphatic transport due to the presence of pituitary tumor. Consistently, our kinetic modeling and regional cerebral tissue quantification revealed significantly lower infusion and clearance rates in all examined regions in rats with spontaneous pituitary tumor than in non-tumor rats, representing a suppressed glymphatic transport in the brain with pituitary tumor.

**Conclusion:**

Our study demonstrates the compromised glymphatic transport in the rat brain with spontaneous pituitary tumor. The reduced efficiency in cerebral waste clearance increases the risk for neurodegeneration in the brain that may underlie the cognitive impairment commonly seen in patients with pituitary tumors.

## 1. Introduction

As common intracranial neoplasms, pituitary tumors induce a broad range of adverse changes in the brain (1, 2). In addition to a mass effect on nearby brain tissue and cranial nerve structures (3, 4), pituitary tumor induced endocrine dysfunction plays an important role in affecting brain function (5–7). Cumulative data demonstrate that hormonal disorders caused by pituitary tumors contribute to structural alterations (7–10), metabolic abnormalities (11–14) and cerebrovascular disease (15–18) that are accompanied by a wide variety of adverse neuropsychological symptoms and neurocognitive consequences (1, 19–22) in patients. Although the underlying mechanisms leading to neurocognitive deficits in patients with pituitary tumors remain to be fully elucidated, growing evidence suggests that aberrant hormone secretion may be a major factor for cognitive impairment (11, 22, 23). However, while the abrogation of abnormal hormone level is associated with the improvement of cognitive function (21, 23, 24), incomplete recovery from structural alterations (6, 7, 25) and partial restoration from cognitive deficits with diverse courses (6, 26, 27) are observed in patients with normalized hormone level after successful treatment. Moreover, cerebrovascular pathology (e.g., cerebral infarction) arising from non-functioning pituitary tumor is independent of hormone replacement therapy (28), and cerebrovascular mortality remains elevated in the treated patients regardless of post-treatment hormone levels (18, 29). These findings suggest that tumor-induced deleterious impact on the central nervous system (CNS) and cerebral vasculature that are linked to the decline of cognitive function (17, 30–32) persists after resolution of hormone abnormalities, and at the same time, indicate that factors other than hormone imbalance, that act particularly upon the cognitive impairment, are likely at work.

The glymphatic system is a brain-wide peri-vascular network, involving cerebrospinal fluid (CSF) recirculation throughout the brain and facilitation of interstitial solute clearance from the CNS (33, 34). The glymphatic transport consists of peri-arterial influx and peri-venous efflux supported by astrocytic aquaporin-4 (AQP4) water channels (35). Dysfunction of glymphatic waste clearance results in accumulation of neurotoxic products within the brain parenchyma, leading to neurodegeneration and corresponding neurocognitive deficits (36–38). Pituitary tumors have been shown to cause systemic comorbidities such as cardiovascular and cerebrovascular disease that provoke diffuse and sustained impact on the brain (16–18, 39). The vascular pathologies associated with these comorbidities may disturb the glymphatic waste clearance which in turn, alters the neurocognitive performance. To our knowledge, however, little is known about the status of glymphatic function in the brain in the presence of this type of intracranial neuroendocrine tumor. Investigation of the alterations of glymphatic transport concurrent with the appearance of pituitary tumor may provide insight into the mechanisms underlying cognitive disorders widely experienced by patients and may facilitate development of treatment strategies for amelioration of cognitive impairment.

Glymphatic transport is characterized and monitored using intracisternal administration of CSF tracers, and the glymphatic function is evaluated by the kinetic features of the surrogate CSF tracer “waste” solutes as they pass through the brain (40–43). As an *in vivo* non-invasive tool, magnetic resonance imaging (MRI) plays an important role in revealing the glymphatic transport routes by visualizing spatiotemporal CSF tracer trajectory (42, 44) and in quantifying the glymphatic transport function by using tracer-induced dynamic signal information (40, 45–47). With dynamic contrast-enhanced MRI (DCE-MRI), impaired glymphatic function has been detected in a wide range of neurological diseases in both the human (48, 49) and animal (50–53) studies. Using DCE-MRI and our advanced kinetic modeling, we have demonstrated that the glymphatic transport in the rat brain is suppressed under conditions of diabetes, traumatic brain injury and aging (41, 54, 55). With MRI and our corresponding analyses, the aim of present study is to investigate whether and how the glymphatic function changes in rats carrying spontaneous pituitary tumor. We then demonstrate that the presence of a pituitary tumor compromises glymphatic transport and thereby may contribute to the cognitive impairment commonly observed in patients with pituitary tumor.

## 2. Materials and methods

All experimental procedures were approved by the Institutional Animal Care and Use Committee of Henry Ford Health and carried out in accordance with the NIH Guide for the Care and Use of Laboratory Animals.

### 2.1. Animals and experimental procedures

Although tumors arising in the pituitary gland are found in both sexes at a wide range of ages, accumulating clinical (2, 56) and laboratory (57–59) data show that females are more likely to develop these tumors than males, and that the incidence rate of these neuroendocrine tumors increases with advancing age (60, 61). We, therefore, first focused on aged female rats in our experimental investigation.

Rats (22–24 months, female, Wistar, Charles River, Wilmington, MA, United States) with and without spontaneously occurring pituitary tumor (*n* = 7/per group) were subjected to the identical experimental procedures, including the surgical preparation for contrast administration into the cisterna magna, and subsequent MRI measurements.

Catheter implantation (62) was performed for each animal before the MRI scan. Briefly, the rats were initially anesthetized with inhalation of 3% isoflurane and maintained in the range of 1.0–1.5% isoflurane in a mixture of N_2_O (70%) and O_2_ (30%) *via* a nose mask throughout the surgical period. Rectal temperature was controlled at 37°C ± 1°C using a feedback-regulated water heating system. The head of the anesthetized rat was mounted in a stereotactic frame with care to permit spontaneous breathing. After the Atlanto-occipital membrane was exposed using a midline dorsal neck incision, a polyethylene catheter (PE-10 tubing; Becton Dickinson, MD, United States) filled with saline was inserted into the subarachnoid cisterna magna space *via* a small durotomy made with a 27gauge needle. The outside part of catheter was fixed onto the occipital bone with superglue and the skin incision was closed around the catheter.

Magnetic resonance imaging was performed with a 7 T system (Bruker–Biospin, Billerica, MA, United States) (62). A birdcage type coil was used as the transmitter and a quadrature half-volume coil as the receiver. The animal with catheter implantation was securely fixed on a MR-compatible holder equipped with an adjustable nose cone for administration of anesthetic gases and stereotaxic ear bars to immobilize the head. For reproducible positioning of the animal in the magnet, a fast-gradient echo imaging sequence was used at the beginning of each MRI session. During image acquisition, anesthesia was maintained by a gas mixture of N_2_O (70%) and O_2_ (30%) with 1.0–1.5% isoflurane (Piramal Inc., Bethlehem, PA, United States), and rectal temperature was kept at 37 ± 1°C using a feedback controlled air heating blower (Rapid Electric, Brewster, NY, United States).

Pituitary tumor provokes changes in signal intensity on MR images (63–66), and is often accompanied by volumetric and morphological alterations of the pituitary gland (65–67). To detect abnormal tissue (e.g., pituitary tumor) within the affected pituitary gland and calculate lesion volume (e.g., tumor size), multi-slice coronal T2-weighted imaging (T2WI; TE = 15, 30, 45, 60, 75, and 90 ms, TR = 4 s, FOV = 32 × 32mm^2^, matrix = 256 × 256, thickness = 1 mm, 15 slices) was performed. To monitor the dynamic influx and clean-out process, 3D T1-weighted imaging (T1WI; TE = 4 ms, TR = 18 ms, flip angle = 12°, FOV = 32 × 32 × 16 mm^3^, matrix = 256 × 192 × 96) with contrast agent of Gd-DTPA was acquired. The time series of T1WI scanning continued for 6 h, starting with three baseline scans followed by intra-cisterna magna (ICM) Gd-DTPA (21 mM concentration) delivery at a constant infusion rate of 1.6 μL/min over 50 min (41, 62) *via* the indwelling catheter connected to a 100 μL syringe (Hamilton Robotics, Reno, NV, United States) mounted on an infusion pump (Harvard Apparatus, Holliston, MA, United States).

### 2.2. Magnetic resonance imaging data processing

The detailed procedures for DCE-MRI data processing and parametric map generation have been previously described (40). To correct for motion that occurs during the 6-h scan, the entire set of sequential images for each animal were co-registered to its initial volume. Then, 3D T1WIs for all animals were co-registered to a standard reference template so that the comparison between groups will be carried out in the common spatial space. With the changes of MRI signal that correspond to the time trajectories of CSF tracer concentrations, brain voxels were clustered into similar regions based on their dynamic responses to the infusion of contrast agent. We chose the derivative of the time-signal-curves (TSCs) as the similarity criterion in order to cluster the voxels based on the tracer dynamics. In a hierarchical clustering scheme, the k-means clustering algorithm splits the voxels into two clusters. Then, it sequentially splits each of the resulting clusters into two new sub-clusters if a described criterion (40) is met (Details of this method are provided in Davoodi-Bojd et al. (40)). TSC for each cluster that represents the retention of infused tracer as a function of time in the tissue cluster was obtained, yielding the required information for our advanced kinetic modeling. Using a defined approach with specific criteria, a local input function (IF) for any formed cluster was found among the TSCs of its neighboring clusters. Compared to the previous modeling with IF obtained from the TSC of whole brain (45), our local IF selected for each cluster is an advance by which the errors arising from the global IF are largely reduced. For each tissue cluster, the parameters characterizing the kinetics of tracer uptake and clearance were derived from its own TSC. Herein, infusion rate is defined by the rate of signal increase from the point immediately after three baseline scans to the peak in the accumulation phase of the TSC, while clearance rate is defined by the rate of signal decrease from the peak to the end of experiment in the relaxing phase of the TSC. After calculating these kinetic parameters in each cluster from its average TSC, parametric maps of infusion rate and clearance rate for whole brain were then generated.

### 2.3. Quantification and statistical analysis

Pituitary tumor was detected by T2WI. Using ImageJ (https://imagej.nih.gov/ij/, ImageJ1.51j8) (68), the abnormal tissue areas in the pituitary gland on contiguous coronal T2WI slices were measured. The T2WI-detected lesion volume (tumor size) was then calculated by adding all the abnormal tissue areas on individual slices and multiplying the total by the slice thickness.

**Figure 1:**
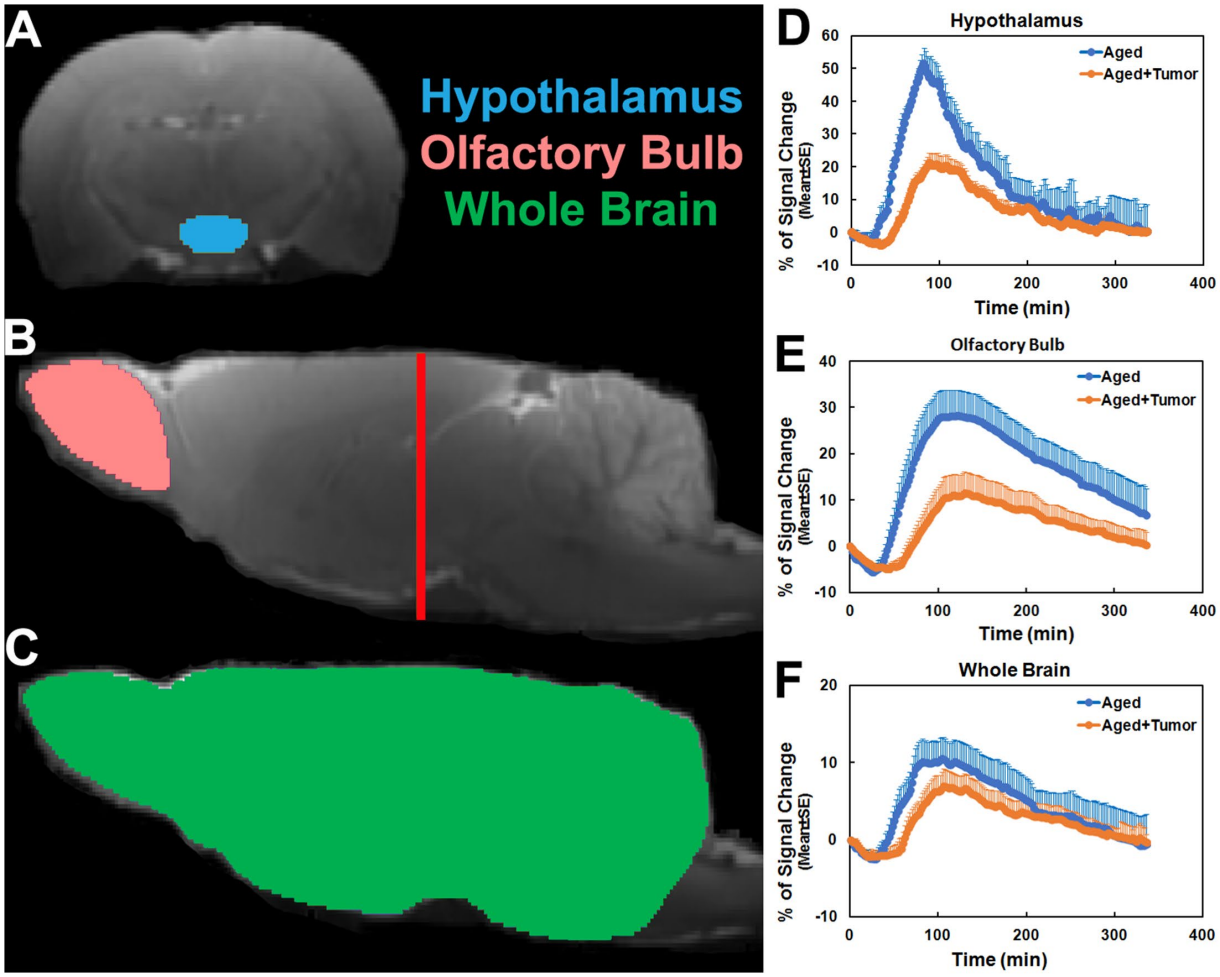
Regions of interest (ROIs; colored anatomical areas in **A**, **B**, and **C**) on the coronal (**A**, Bregma − 3.00 mm) and sagittal (**B**, **C**, Lateral 0.40 mm) sections, and group time-signal-curves (TSCs; **D–F**) obtained from the corresponding ROIs. The red line in **B** indicates the location of the selected coronal section **(A)** on which the ROI of hypothalamus was created. ROIs of olfactory bulb and whole brain were made in two hemispheres of the brain symmetrically. Compared to the rats with normal appearance of pituitary gland, the TSCs measured from the rats with pituitary tumor were characterized by later time points for initial signal increase, longer periods of time before the peak values, and lower percentages of signal change **(D–F)**.

To evaluate the kinetic features of contrast agent transport *via* the glymphatic system within the brain, regions of interest (ROIs) encompassing representative brain tissue areas (such as hypothalamus, olfactory bulb and whole brain) were created on the fixed coronal and sagittal sections of 3D T1WI (Figure 1). With these ROIs, regional TSCs were obtained and compared between groups. Based on these ROIs, evaluations were also conducted on the parametric maps. Group TSCs and parametric measurements for each ROI were presented as mean ± standard error (SE). To detect the effects of pituitary tumor on regional TSCs and on glymphatic transport function characterized by the kinetic parameters, a two-sample *t*-test was performed between groups with *p* < 0.05 inferred for statistical significance. Fisher’s exact test (statistical significance: *p* < 0.05) was employed to compare the difference in proportions of MRI-detected adverse events (i.e., partially enhanced circle of Willis, absence of enhancement in glymphatic influx nodes) between groups.

## 3. Results

### 3.1. Changes of regional TSCs in the rats with pituitary tumor

As shown in Figure 1, the TSCs obtained from the same brain regions differed between groups (Figures 1D–F). Compared to the rats with normal appearance of pituitary gland, the TSCs measured from the rats with pituitary tumor were characterized by delayed time points for initial signal increase, prolonged periods of time before the peak values, and reduced percentages of signal change for all examined brain regions. Consequently, a slower pace for both signal increase before the peak values and signal decrease after the peak values in these brain regions were present in the rats with pituitary tumor than in the rats without pituitary tumor, indicating the retarded influx and clean-out of CSF tracer *via* the glymphatic system. The details of statistical comparison of group TSCs are provided in Table 1, and significant differences in the features of TSCs that represent the glymphatic function were found between groups. These results demonstrate that with pituitary tumor, a reduced glymphatic transport and reduced amount of CSF passing through the glymphatic system are present in the brain.

**Table 1:** Group comparisons of time-signal-curves (TSCs) measured from the examined brain regions (mean ± SE).

	Hypothalamus		Olfactory bulb		Whole brain	
	Aged	Aged + Tumor	Aged	Aged + Tumor	Aged	Aged + Tumor
Time (min) before signal increase	36.50±3.86	56.00±3.70*	44.89±5.27	73.00±7.59*	43.75±5.43	62.00±5.74*
Time (min) before peak value	78.50±4.79	102.29±6.49*	117.75±4.23	138.80±7.66*	104.86±5.52	112.57±5.85
Peak value (% of signal change)	54.25±5.13	20.10±3.25*	28.25±4.68	11.96±4.25*	11.22±2.72	8.74±1.32
Signal accumulation (% of increase/min)	0.69±0.02	0.20±0.03*	0.24±0.05	0.10±0.04	0.13±0.03	0.08±0.02
Signal attenuation (% of decrease/min)	0.24±0.04	0.12±0.01*	0.11±0.02	0.08±0.02	0.08±0.02	0.05±0.004

* *p* < 0.05, aged rats with pituitary tumor vs. aged rats with normal appearance of pituitary gland in the same brain region.

### 3.2. Impairment of glymphatic pathway in the rats with pituitary tumor

As verified with TSCs, our image data showed a lower incidence of contrast-induced enhancement in both pituitary (71 vs. 100%) and pineal (57 vs. 86%) recesses, the glymphatic influx nodes (42, 44), in the rats with pituitary tumor than in the rats without pituitary tumor.

**Figure 2:**
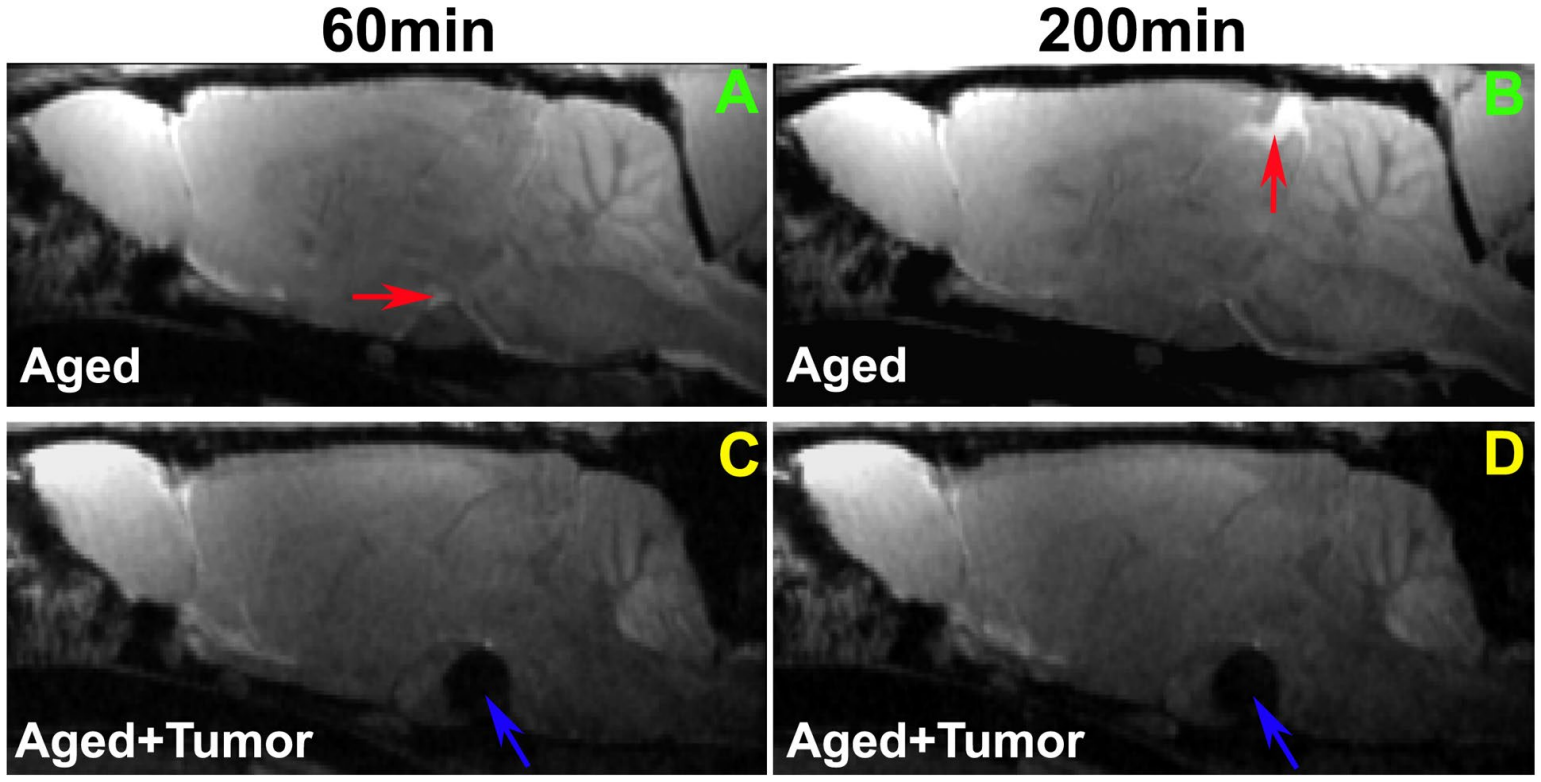
Comparison of enhancement in pituitary and pineal recesses (glymphatic influx nodes) between the representative rats with normal appearance of pituitary gland **(A,B)** and with pituitary tumor (**C,D**; Tumor: indicated by blue arrow). While the enhancement in the pituitary recess (red arrow in **A**) at the earlier time point (e.g., 60 min) and in the pineal recess (red arrow in **B**) at the later time point (e.g., 200 min) after intra-cisterna magna (ICM) contrast injection was observed in the rat brain without pituitary tumor, this enhancement in both pituitary (**A** vs. **C**) and pineal recesses (**B** vs. **D**) was not detected in the rat brain with pituitary tumor. Worthy of note, is the mass effect of pituitary tumor on adjacent gland tissue and brain structures **(C,D)**.

A comparison of representative rats from two groups is shown in Figure 2. While the enhancement in pituitary recess (red arrow in Figure 2A) at the earlier time point (e.g., 60 min) and in pineal recess (red arrow in Figure 2B) at the later time point (e.g., 200 min) after ICM contrast injection was observed in the rat brain without pituitary tumor, this enhancement in both pituitary (Figure 2A vs. Figure 2C) and pineal (Figure 2B vs. Figure 2D) recesses was not detected in the rat brain with pituitary tumor. The lowered incidence of enhancement in the glymphatic influx nodes in rats with pituitary tumor indicates a reduced efficiency in glymphatic transport.

**Figure 3:**
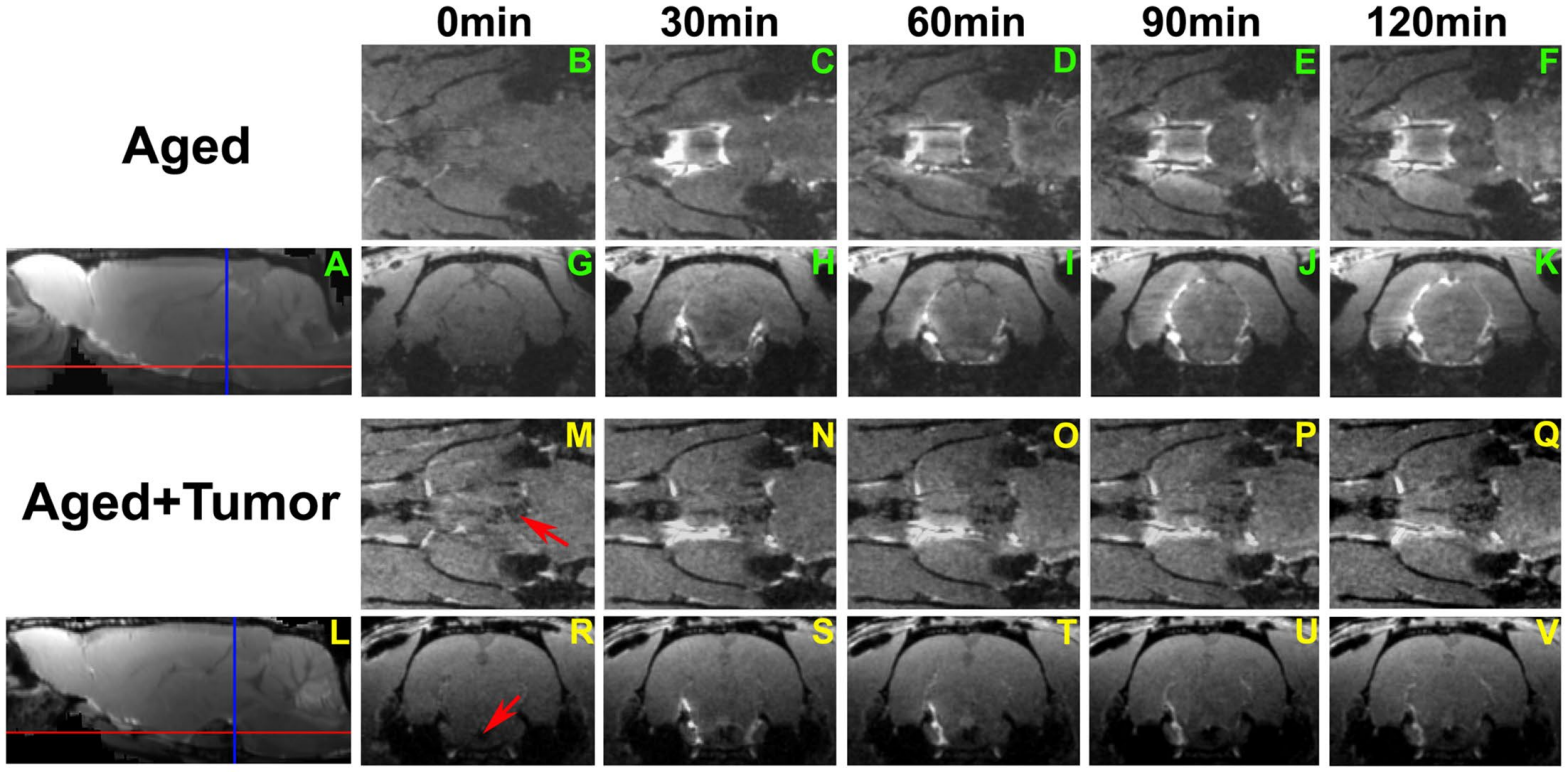
A series of 3D images showing the transport of ICM-injected contrast agent in two representative rat brains with normal appearance of pituitary gland **(A–K)** and with pituitary tumor (**L–V**; Tumor: indicated by red arrow in **M** and **R**). Red and blue lines in **A** and **L** indicate the locations of axial and coronal images that cut across the circle of Willis **(B–F, M–Q)** and pineal recess **(G–K, R–V)**, respectively. Compared to the rat without tumor, partially enhanced circle of Willis (**N–Q** vs. **C–F**) was found in the rat with pituitary tumor. Enhancement in the pineal recess occurred between 90 min and 120 min **(J–K)** for the rat without tumor, while this contrast-induced enhancement was not detected during the same period **(U–V)** for the rat with tumor. This result was reflected correspondingly on the co-registered mean sagittal images **(A,L)**, with the pineal recess enhancement present on one image **(A)** but not on the other **(L)**.

A series of 3D images (Figure 3) further revealed a partially enhanced circle of Willis (Figures 3N–Q vs. Figures 3C–F) in a representative rat with pituitary tumor, suggesting that the peri-arterial influx was blocked in part. It is worth noting that concurrent with this blockage was the absence of pineal recess enhancement in the brain (Figures 3J–K vs. Figures 3U–V; Figure 3A vs. Figure 3L). These dynamic image data demonstrate an impaired glymphatic pathway and the corresponding impeded glymphatic transport. A significantly larger proportion of partially enhanced circle of Willis was found in the rats with pituitary tumor than in the rats without pituitary tumor (86 vs. 14%, *p* < 0.033), indicating an association between the occurrence of pituitary tumor and disrupted peri-vascular pathway in the circle of Willis.

### 3.3. Reduction of glymphatic transport in the rats with pituitary tumor

**Figure 4:**
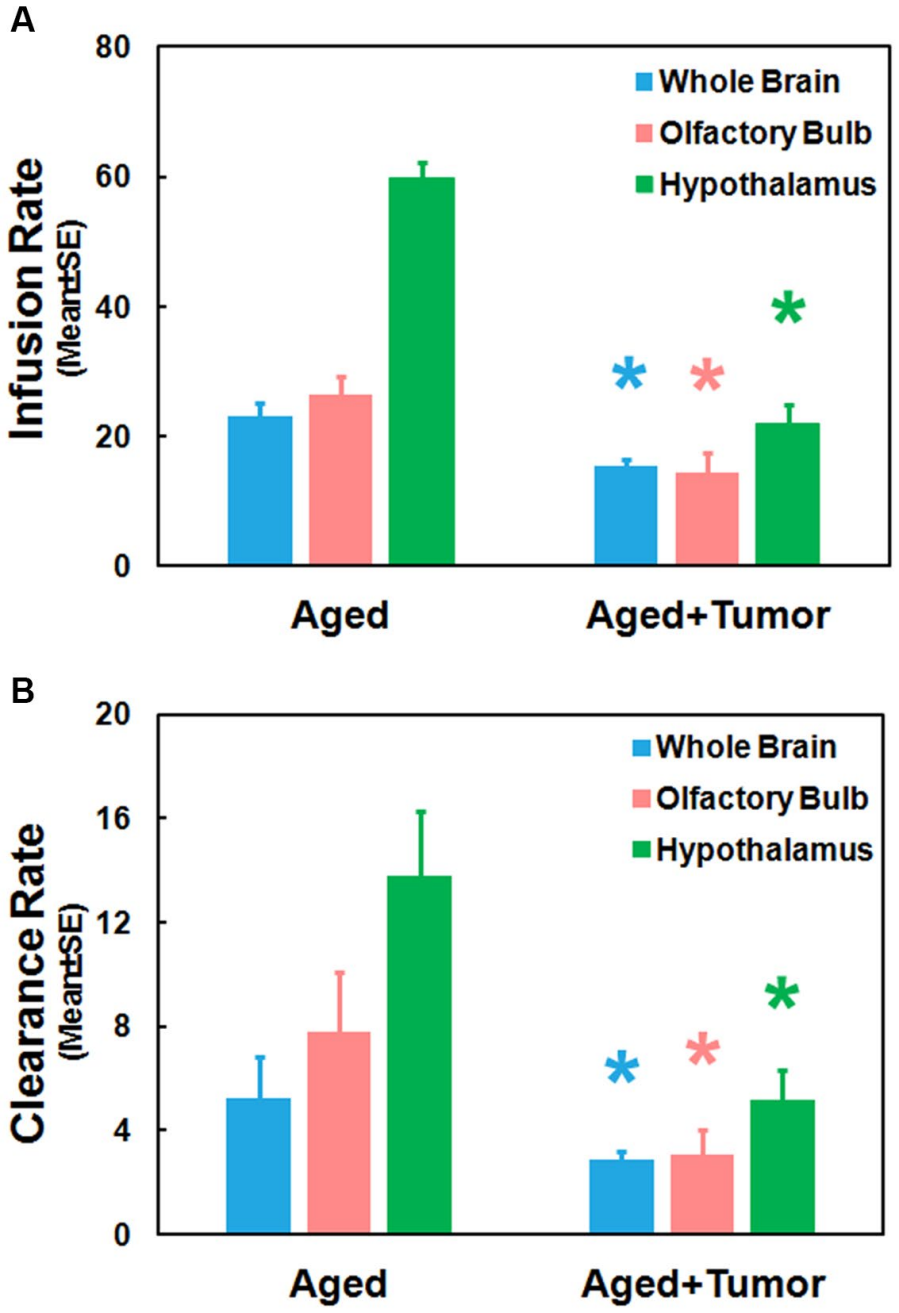
Group comparisons of glymphatic transport function characterized by kinetic parameters measured in the examined brain regions. Significantly reduced infusion rate **(A)** and clearance rate **(B)** in these brain regions were detected in the rats with pituitary tumor compared to the rats without pituitary tumor. ^*^p < 0.05, aged rats with pituitary tumor vs. aged rats with normal appearance of pituitary gland in the same brain region.

Figure 4 provides the group comparison of infusion and clearance rates, the important model-derived kinetic parameters that characterize the glymphatic transport function. Significantly reduced infusion rate (Figure 4A; Whole Brain: 15.49 ± 0.97 vs. 22.99 ± 2.26, *p* < 0.01; Olfactory Bulb: 14.42 ± 3.10 vs. 26.51 ± 2.75, *p* < 0.03; Hypothalamus: 22.04 ± 2.97 vs. 59.75 ± 2.45, *p* < 0.001) and clearance rate (Figure 4B; Whole Brain: 2.86 ± 0.32 vs. 5.26 ± 1.55, *p* < 0.05; Olfactory Bulb: 3.04 ± 0.95 vs. 7.77 ± 2.28, *p* < 0.03; Hypothalamus: 5.19 ± 1.10 vs. 13.79 ± 2.48, *p* < 0.003) in the examined brain regions were found in the rats with pituitary tumor compared to the rats with normal appearance of pituitary gland. Consistent with our imaging observations (Figures 2, 3), these kinetic quantifications with statistical differences between groups demonstrated a compromised glymphatic transport in the rat brain with pituitary tumor.

### 3.4. Effect of pituitary tumor size

Our image data showed that the bigger pituitary tumor size, the higher incidence of unfavorable changes in the brain. Despite the presence of a partially enhanced circle of Willis (14%) and the absence of enhancement in glymphatic influx nodes (pineal recess: 14%; pituitary recess: 0%) in the rats without pituitary tumor, much higher proportions of these adverse outcomes were observed in the rats with pituitary tumor (86, 43, and 29%, respectively). In the tumor group, the majority of the cases with these consequences were found in the rats with tumor size larger than 3.4mm^3^. Correspondingly, our image data showed that a bigger tumor size was associated with a greater mass effect on the surrounding gland tissue and brain structures (Figure 2).

## 4. Discussion

Using DCE-MRI and mathematical modeling, we investigated the changes of glymphatic transport in the aged rats carrying spontaneous pituitary tumor. The current study demonstrated that with pituitary tumor, a reduced efficiency in glymphatic transport function was present in the brain. We, for the first time, provide MRI evidence of concurrently impaired glymphatic pathway and impeded glymphatic transport in the aged rat brain, exacerbated by the presence of pituitary tumor. Consistent with these imaging observations, our kinetic modeling and regional quantification revealed a more severe suppression of glymphatic transport in the rats with pituitary tumor than in the rats with normal appearance of the pituitary gland.

As depicted by the trajectory of ICM-injected CSF tracer on MRI (i.e., spatiotemporal pattern of contrast-induced enhancement), previous studies demonstrate that the glymphatic system moves solute from the subarachnoid space of the cisterna magna into the brain parenchyma *via* key glymphatic transport pathways (42, 44). The solute flows along the ventral surface of the brain and then towards the areas of olfactory bulb and pineal gland with the pituitary and pineal recesses serving as the influx nodes. Hypothalamus is directly connected to pituitary gland by the pituitary stalk (69). This specific brain region, therefore, seems more likely to be impacted by the gland pathology (e.g., pituitary tumor). Moreover, hypothalamus is immediately adjacent to the influx node of pituitary recess, while olfactory bulb is directly associated with the glymphatic transport pathway (42, 44). Regarding the glymphatic influx and efflux, both hypothalamus and olfactory bulb could be the sensitive brain regions which exhibit the changes in glymphatic transport, if any, due to the occurrence of pituitary tumor. To detect both regional and global alterations, we therefore selected these representative structural areas as well as whole brain as the regions of interest (ROIs) and evaluated the glymphatic transport function.

The TSCs measured from these examined ROIs (Figures 1D–F; Table 1) showed a reduced efficiency in glymphatic transport characterized by a slower pace for both signal accumulation and signal attenuation in the rats with pituitary tumor than in the rats with normal appearance of pituitary gland. Consistently, our dynamic images revealed the obstruction of peri-vascular CSF flow concomitant with the absence of pineal recess enhancement in the tumorous rat (Figure 3), representing the impaired glymphatic pathway and retarded glymphatic transport. The partially blocked peri-vascular flow (14%) and non-enhanced pineal recess (14%) were detected in non-tumor rats probably owing to aging-related glymphatic deterioration (70–72). The incidence of these adverse events, however, was much higher in rats with pituitary tumor (86 and 43%, respectively), indicative of a detrimental effect of pituitary tumor on the peri-vascular pathway and glymphatic transport function. Consequently, such an intensification of disrupted glymphatic system may further increase the burden of harmful metabolites and proteins (e.g., amyloid-β, tau) (34, 36, 38, 43) in the brain known to negatively affect the cognitive status (41, 43, 73).

Compared to rats without pituitary tumor, a higher incidence of impaired peri-vascular pathway in rats with tumor may be attributed to a higher prevalence of cerebrovascular disease associated with pituitary tumor (15, 16, 18). By causing abnormal hormone levels (74, 75) and metabolic derangements (5, 12, 76), pituitary tumor poses an excess risk of unfavorable alterations in the cerebrovascular network, structurally and functionally affecting both large and small vessels. The elevated cerebrovascular events include arteriosclerosis and atherosclerosis pathologies, high blood pressure, cerebral infarcts and cerebral microbleeds (17, 18, 77, 78) that diffusely disturb the proper functioning of vascular and peri-vascular transport. In line with these well-recognized changes in the cerebrovascular system, our image data revealed an occluded glymphatic influx pathway (e.g., partially enhanced circle of Willis; Figure 3) that obstructed CSF movement, representing damaged peri-vascular transport along the large arterial vessel. As captured by dynamic images and evaluated by kinetic parameters of infusion and clearance rates, this blockage of peri-arterial influx was not only accompanied by the impeded CSF flow to the glymphatic influx node (Figure 3), but also coincided with the markedly reduced pace of CSF circulation throughout the brain (Figure 4), suggesting the involvement of both large and small cerebral vessels. In addition to the cerebrovascular pathologies that provoked systemic and durable impact on the glymphatic transport, our data also demonstrated that a mass effect (3, 4) of pituitary tumor on adjacent structures, capable of injuring the vasculature (79–81), was a factor that could not be excluded. Our MRI data indicate that the pituitary tumor presses on the surrounding gland tissue and brain structures (Figure 2). The bigger the tumor size, the more severe the compression and injury of the surrounding areas. The mass effect of a large tumor may contribute to the higher incidence of damaged peri-vascular space (Figure 3) and retarded CSF flow (Figure 2). Thus, the pituitary tumor induced dysfunction of peri-vascular transport provokes the compromised cerebral waste clearance (Figure 4B), likely underlying the neurocognitive impairment (36–38).

Previous investigations demonstrated a higher incidence of intracranial aneurysms in patients harboring pituitary tumor than in the general patient population (82–84), with the circle of Willis being the most common location for aneurysm (83, 85). The increased incidence of aneurysms coexisting with pituitary tumors represents the tumor-induced degenerative modification of vessel wall (85–88). Adding to the present understanding of vascular alterations in the circle of Willis, the current study provides new evidence of impaired peri-vascular transport associated with the pituitary tumor in this important vascular structure (Figure 3). With the pituitary tumor, the circle of Willis appears as a particularly noteworthy site where the vascular and peri-vascular changes are manifest and detectable.

Due to the following limitations, the results obtained from the present study should be interpreted with caution. First, the number of animals in the experimental groups was small. Although the animals were approximately the same age, the spontaneous pituitary tumors carried by individual animals were at different stages and/or status (e.g., microadenomas, macroadenomas, and invasiveness). Thus, further investigations of the effects of tumor status on the glymphatic measures with additional animals and specific subgroups are warranted. Secondly, we recruited rats with pituitary tumor based on MRI detection, and therefore, both functioning and non-functioning pituitary tumors might be included. Regarding the vascular factors that mainly impact the glymphatic transport, nevertheless, cerebrovascular pathologies associated with either functional or non-functional tumors appeared not related to hormone levels (18, 28, 29). Extended study with diagnostic tests, such as blood and urine tests of hormone levels, would provide information about the effects of tumor types on the alterations of glymphatic function. Finally, the differences between the human and the animal in anatomical and other aspects should be kept in mind when applying the animal experimental results to the human. Olfactory bulb, occupying a large portion of the rat brain but a small portion of the human brain, is an example, although for both the human (89, 90) and the rodents (90–92), it serves as major efflux route for brain waste clearance.

In summary, this is, to our knowledge, the first study showing that suppressed glymphatic transport is present in the brain with spontaneous pituitary gland tumor. The impaired glymphatic pathway associated with the occurrence of pituitary tumor contributes to the reduced efficiency in glymphatic transport which in turn, suggests an increased risk for neurodegeneration in the brain. Our results, therefore, suggest an underlying role of glymphatic dysfunction in cognitive impairment commonly seen in patients with pituitary tumors.

## Data availability statement

The original contributions presented in the study are included in the article/Supplementary material, further inquiries can be directed to the corresponding author.

## Ethics statement

The animal study was reviewed and approved by the Institutional Animal Care and Use Committee of Henry Ford Health.

## Author contributions

LL wrote the manuscript and performed MRI data processing and analysis. GD and QL performed MRI experiments, data analysis and interpretation. LZ and HL conducted the specific surgery for MRI experiments and data acquisition. ED-B performed MRI data modeling. MC, ZZ, and QJ contributed to conception and design of the study, manuscript revision. All authors contributed to the article and approved the submitted version.

## Funding

This work was supported by grants from National Institutes of Health (NIH): RF1 AG057494 (QJ and LZ), RO1 NS108463 (QJ).

## Conflict of interest

The authors declare that the research was conducted in the absence of any commercial or financial relationships that could be construed as a potential conflict of interest.

## Publisher’s note

All claims expressed in this article are solely those of the authors and do not necessarily represent those of their affiliated organizations, or those of the publisher, the editors and the reviewers. Any product that may be evaluated in this article, or claim that may be made by its manufacturer, is not guaranteed or endorsed by the publisher.
